# Functional Role of *AsAP* in the Reproduction of *Adelphocoris suturalis* (Hemiptera: Miridae)

**DOI:** 10.3390/insects13080755

**Published:** 2022-08-22

**Authors:** Shidong Qin, Bangqin Zhu, Xingxing Huang, J. Joe Hull, Lizhen Chen, Jing Luo

**Affiliations:** 1 State Key Laboratory of Biocatalysis and Enzyme Engineering, School of Life Sciences, Hubei University, Wuhan 430062, China; 2 Hubei Insect Resources Utilization and Sustainable Pest Management Key Laboratory, College of Plant Science and Technology, Huazhong Agricultural University, Wuhan 430070, China; 3 Guiyang Center for Disease Control and Prevention, Guiyang 550003, China; 4 Pest Management and Biocontrol Research Unit, US Arid Land Agricultural Research Center, USDA Agricultural Research Services, Maricopa, AZ 85138, USA

**Keywords:** *Adelphocoris suturalis* Jakovlev, aspartic protease, reproduction, ovarian development, RNAi

## Abstract

**Simple Summary:**

*Adelphocoris suturalis* is a destructive pest that targets > 270 plants, including cotton, maize, soybean and fruit trees, which has caused tremendous crop losses worldwide. Considering the pesticide resistance and safety issues that are caused by chemicals and the readiness of RNAi technology for use in pest control, the identification of a candidate gene for RNAi remains an active area of research that deserves more attention. Reproduction is the basis for the survival of a species and a targeted disruption to this process would efficiently regulate its population; however, this has not yet been extensively investigated in *A. suturalis*. In this study, we first isolated a putative *A. suturalis* *aspartic protease* (*AsAP*) gene. The role of *AsAP* in the reproduction of the pest was subsequently illuminated using the dsRNA-mediated knockdown method. The findings expanded our understanding of *A. suturalis* reproductive development and provided a potential candidate target gene for the development of RNAi-based pest control strategies.

**Abstract:**

*Adelphocoris suturalis* Jakovlev (Hemiptera: Miridae) is an omnivorous agricultural pest that has severe economic impacts on a diverse range of agricultural crops. Although the targeted disruption of reproductive development among insects has been proposed as a novel control strategy for pest species, the current understanding of the physiology and molecular mechanisms of *A. suturalis* reproduction is very limited. In this study, we isolated a putative *A. suturalis* *aspartic protease* (*AsAP*) gene that is highly expressed in the fat body and ovaries of sexually mature females. The double-stranded RNA (dsRNA)-mediated knockdown of *AsAP* suppressed ovarian development and negatively impacted female fertility, which suggested that it plays an essential role in *A. suturalis* reproduction. The results of this study could help to expand our understanding of *A. suturalis* reproductive development and have the potential to facilitate the development of effective strategies for the better control of this pest species.

## 1. Introduction

Reproductive capacity is essential for the continued existence of a species over time and is, in part, one of the reasons that insects are among the most successful organisms on the planet in terms of sheer numbers. Most insects are characterized by large egg production (100–500 eggs over a lifetime), high egg hatching rates and short life cycles (often 2–4 weeks), which enable them to produce remarkably large numbers of offspring. For example, pairs of blow flies produce an average of 144 progeny: half of which are male and half of which are female. Theoretically, one pair of flies could produce 10,368 offspring by the third generation and 746,496 by the fourth generation. At this rate, the fly population could grow to more than 1 × 10^17^ individuals across 10 generations within a single year [1]. This remarkable capacity is one reason that pest outbreaks often develop suddenly. Therefore, the targeted disruption of reproductive development represents a promising strategy for pest control.

Aspartic proteases, which are one of the seven classes of proteolytic enzymes that are characterized by conserved Asp-Thr/Ser-Gly (DT/SG) motifs in active sites, are optimally active at acidic pH values [2,3]. They break down proteins into smaller polypeptides or single amino acids by cleaving the peptide bonds within the proteins [4] and they have varied biological roles in arthropods, including yolk degradation during embryogenesis [5], digestion [6,7] and immune responses against pathogen invasion [8,9]. Boophilus yolk cathepsin, which is an aspartic protease that is expressed in the eggs of *Boophilus microplus,* facilitates the breakdown of vitellogenin for uptake during embryogenesis [10]. The intestinal aspartate protease (TiCatD) of *Triatoma infestans* is strongly induced after feeding and catalyzes hemoglobin digestion in the hosts [7]. BmAP, which is an aspartic protease that is isolated from the midgut of *Rhipicephalus* (*Boophilus*) *microplus*, is responsible for the in vivo production of a hemocidin (a potent antimicrobial peptide) [9].

RNA interference (RNAi) refers to the phenomenon of the post-translational silencing of gene expression that occurs in response to the introduction of double-stranded RNA (dsRNA) into a cell [11]. This cellular response could be exploited to enable the specific inhibition of any target gene expression. Therefore, RNAi has rapidly emerged as a potent research tool in the study of gene function, regulation and molecular interaction [12,13,14]. In addition, RNAi has also shown great potential for use in pest management. When dsRNAs that target critical insect gene(s) are ingested by a pest, the downregulation of these genes by RNAi could result in retarded growth, deformity or even death, which could eventually reduce the population of the targeted pests [15,16].

*Adelphocoris suturalis* Jakovlev (Hemiptera: Miridae) has a broad host range of 270 known species of plants (e.g., cotton, maize, soybean and fruit trees) and is one of the most destructive pests in the major cotton growing regions of China [17]. Pest management approaches currently rely on chemical pesticides; however, prolonged dependence on insecticides can lead to the development of resistance among the target population, in addition to serious environmental and safety issues [18]. As a consequence, alternative eco-friendly approaches are needed and targeted reductions in insect fertility have been proposed as effective control strategies.

Considering the important function of *aspartic protease* genes in the reproduction of insects [19], we screened and identified 42 *aspartic protease* genes from our previous transcriptome data of *A. suturalis* and the gene with the highest expression level among sexually mature females was selected for further study. In our research, we examined the cloning, expression and characterization of the cDNA that encodes *A. suturalis* aspartic protease (*AsAP*) and assessed its in vivo biological function using the RNAi-mediated knockdown method. We found that the *AsAP* gene was highly expressed in the fat body and ovaries of sexually mature females. The downregulation of *AsAP* expression significantly suppressed both ovarian development and female fertility, which indicated that it plays an essential role in *A. suturalis* reproduction. These findings further expanded our understanding of *A. suturalis* reproduction development and suggested that *AsAP* could be a useful candidate target gene for the development of novel *A. suturalis* control strategies.

## 2. Materials and Methods

### 2.1. Insect Rearing

The *A. suturalis* were initially collected from cotton fields around Wuhan City, China. The nymphs were fed on mung bean sprouts whereas and the adults were reared on green beans [20]. All colonies were maintained in climate chambers (Ruihua PH250GS, Wuhan, China) at 26 ± 2 °C with a 70–80% relative humidity and a 16:8 h light:dark cycle. Newly emerged adults were separated daily and were considered to be 1 day old.

### 2.2. cDNA Cloning and Sequence Analysis

The total RNA was isolated from 8-day-old *A. suturalis* females using the TRIzol reagent (Invitrogen, LifeTechnologies, Carlsbad, CA, USA). The total RNA (1 μg) was reverse transcribed using a PrimeScript™ RT Reagent Kit with a gDNA Eraser (Takara, Kyoto, Japan) and was then employed as a template to amplify fragments of *AsAP*, according to *A. suturalis* transcriptome data [21]. A smart kit for the rapid amplification of cDNA ends (RACE) (Takara, Kyoto, Japan) was used to facilitate the amplification of full-length *AsAP*, according to the manufacturer’s instructions. The resulting product was gel-purified (AxyGen, Suzhou, China), ligated into a pEASY-T1 vector (TransGen, Beijing, China) and sequenced. The resultant sequence information was submitted to the National Center for Biotechnology Information (accession number: OP019357). All primers that were used are indicated in Table 1.

The *AsAP* amino acid sequence was deduced using the ExPASy Translate tool, the putative functional domains were predicted using the EMBL SMART program [22], the theoretical molecular weights were determined using PeptideMass [23] and the putative asparagine-linked glycosylation sites were determined using NetNGlyc 1.0 [2]. Our phylogenetic analyses were conducted based on the neighbor-joining method and a Jones–Taylor–Thornton substitution in MEGA 7.0 [24].

### 2.3. Reverse Transcription–Quantitative Polymerase Chain Reaction (RT-qPCR)

The total RNA extraction and cDNA synthesis were performed as described above. The RT-qPCR was performed using a Bio-Rad Detection iQ2 System (Bio-Rad, Hercules, CA, USA) with a SYBR Premix Ex Taq Kit (Takara, Kyoto, Japan), as described previously in [25]. In brief, the reactions were carried out in a final volume of 10 μL, which contained 2 μL of diluted cDNA template, 5 μL of 2 × SYBR^®^ Premix ExTaq ™ II (TaKaRa, Kyoto, Japan) and 400 nmol L ^–1^ of each gene-specific primer (Table 1). The thermocycler parameters were set to 95 °C for 30 s, followed by 40 cycles of 95 °C for 5 s and 62 °C for 30 s. The results were normalized to *elongation factor-1γ* (*EF1γ*) [25] and the relative abundance was determined using the 2^−∆∆CT^ method across at least three independent biological replicates [26,27].

### 2.4. RNA Interference (RNAi) in A. suturalis

Firstly, the sequence of *AsAP* was blasted against the *A. suturalis* transcriptome data and other insect genomic or transcript data from the NCBI. A 613 bp fragment (550–1162 bp) of *AsAP* that had no homologous fragments over 19 consecutive bases with all known insect genes and transcripts was selected as the target sequence of the dsRNA. Then, the dsRNA targeting for *AsAP* (*dsAsAP*) was synthesized using a T7 transcription kit (Fermentas, Lithuania) from PCR-generated templates following a previously described protocol [22]. Similarly, in accordance with the above method, the dsRNA for *green fluorescent protein* (*GFP*) was synthesized (*dsGFP*) and used as the control. The primers that were used to generate the T7 templates are listed in Table 1. Then, 100 nL of dsRNAs (10 μg/μL) was injected into the conjunctives between the metathorax and abdomen of 1-day-old *A. suturalis* females using a micro-injector (World Precision Instruments, Sarasota, FL, USA), which was then collected (*n* = 5) at 5, 10, 14 and 18 days post-injection for the confirmation of the target transcript knockdown. The ovaries of unmated females (*n* > 20) were dissected 10 days post-dsRNA injection to determine the effects on ovarian development and the number of oocytes per ovary pair. Four common reproductive parameters (i.e., pre-oviposition period, lifetime fecundity (total number of eggs laid), adult longevity and egg hatching rate) were evaluated to assess the role of *AsAP* in *A. suturalis* reproductive capacity. The reproductive parameters were analyzed as described previously in [28]. For the egg hatching rate analysis, more than 500 eggs were examined. To determine the pre-oviposition period, lifetime fecundity and adult longevity, more than 30 females from each treatment group were tested. Each experiment was conducted three times.

### 2.5. Data Analysis

Our statistical analyses were conducted using SPSS 24.0 (SPSS Inc., Chicago, IL, USA) and the values were expressed as means ± standard error (SEM). The tissue- and stage-dependent expression profiles of *AsAP* were analyzed using one-way ANOVA, followed by Tukey’s honestly significant difference (HSD) test. Different letters were used to show significant differences (*p* < 0.05). The differences between the *dsGFP* and *dsAsAP* treatments were analyzed using Student’s *t*-tests for unpaired sets. Asterisks were used to indicate statistically significant differences (ns, not significant; *, *p* < 0.05; **, *p* < 0.01; ***, *p* < 0.001). The figures in this paper were drawn using GraphPad Prism 7 (GraphPad Software, La Jolla, CA, USA) and were assembled in either Adobe Illustrator CS6 or Photoshop CS6 (Adobe Systems Inc., San Jose, CA, USA).

## 3. Results

### 3.1. Cloning and Identification of A. suturalis Aspartic Protease (AsAP)

**Figure 1:**
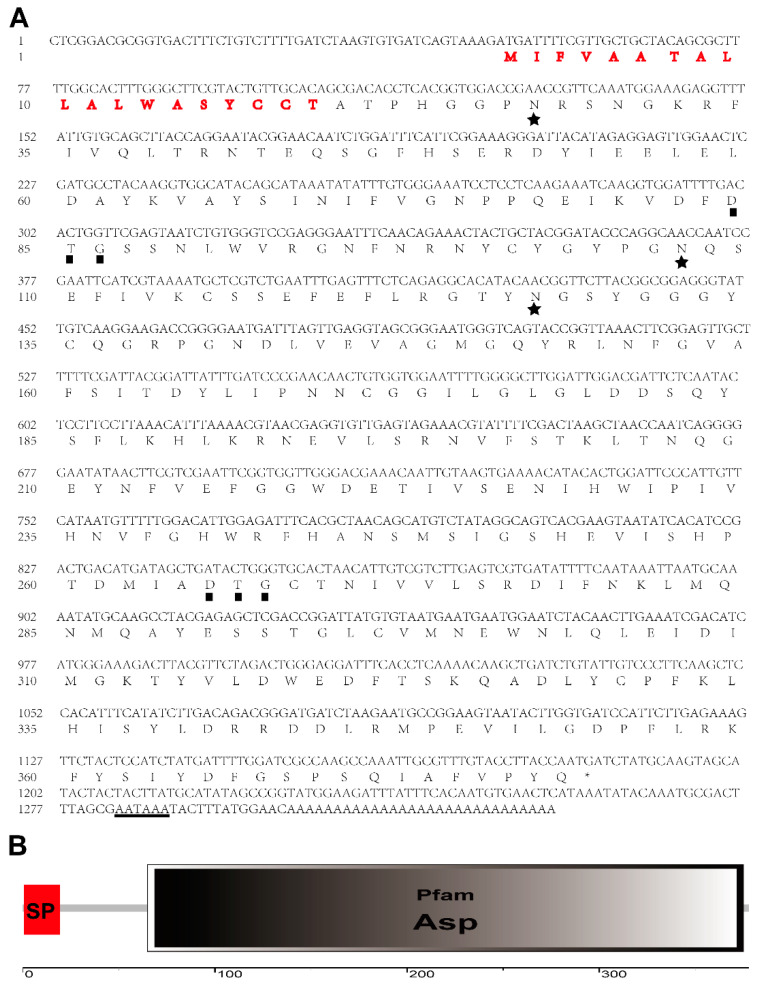
The structural domains and sequence of *Adelphocoris suturalis* aspartic proteases (*AsAP*): (**A**) the nucleotide and deduced amino acid sequence of *AsAP* with the putative signal peptide indicated by red letters, the potential *N*-glycosylation sites indicated by stars, the amino acids (DTG) comprising conserved catalytic sites indicated by solid squares and the putative polyadenylation signal (AATAAA) indicated by the underline; (**B**) a schematic diagram illustrating the functional domains of *AsAP*. SP: signal peptide; Asp: aspartic domain.

**Figure 2:**
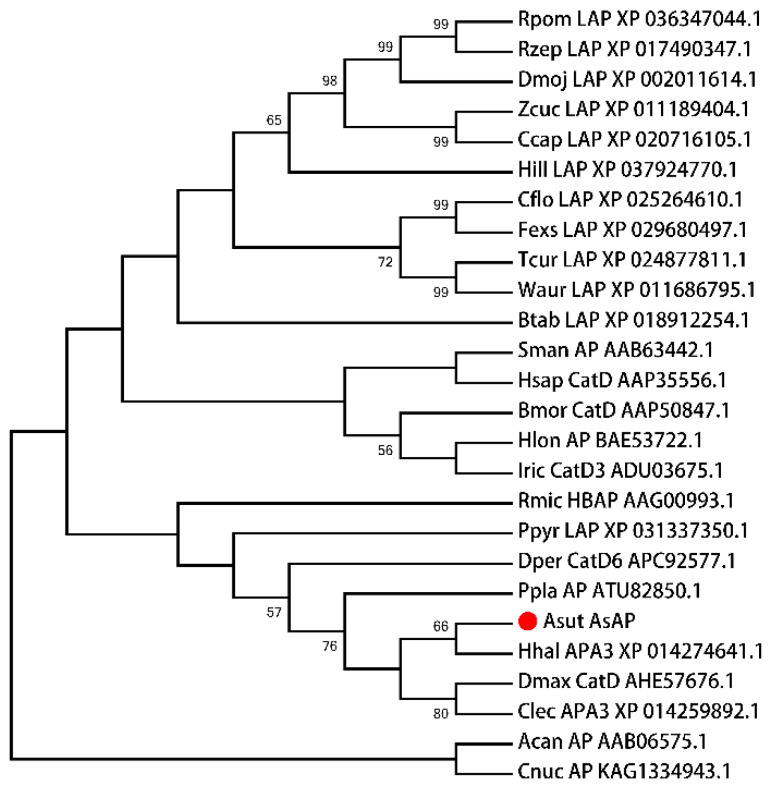
The phylogenetic relationships in the *Adelphocoris suturalis* aspartic protease (*AsAP*). A neighbor-joining tree was constructed using the JTT model for amino acids with confidence values (>50%) shown on the branches, which were based on 1000 rapid bootstrap replicates. Species abbreviations are: Asut, *Adelphocoris suturalis;* Hlon, *Haemaphysalis longicornis*; Ppla, *Pristhesancus plagipennis*; Cflo, *Camponotus floridanus*; Hill, *Hermetia illucens*; Dper, *Dysdercus peruvianus*; Dmoj, *Drosophila mojavensis*; Zcuc, *Zeugodacus cucurbitae*; Ppyr, *Photinus pyralis*; Btab, *Bemisia tabaci*; Fexs, *Formica exsecta*; Rpom, *Rhagoletis pomonella*; Rzep, *Rhagoletis zephyria*; Ccap, *Ceratitis capitate*; Tcur, *Temnothorax curvispinosus*; Waur, *Wasmannia auropunctata*; Dmax, *Dipetalogaster maximus*; Rmic, *Rhipicephalus microplus*; IRic, *Ixodes Ricinus*; Bmor, *Bombyx mori*; Sman, *Schistosoma mansoni*; Acan, *Ancylostoma caninum*; Hsap, *Homo sapiens*; Cnuc, *Cocos nucifera*; Hhal, *Halyomorpha halys*; and Clec, *Cimex lectularius*.

Based on our previous *A. suturalis* transcriptomic data, we used BLASTP against the Uniprot database to obtain 42 transcript fragments of *aspartic protease*. Then, the *A. suturalis* aspartic protease (*AsAP*) with the highest expression level in sexually mature females was chosen for further functional study. The missing sequences (cDNA ends) of *AsAP* were cloned by employing RACE PCR. Finally, we successfully cloned a full-length *AsAP* cDNA (accession number: OP019357). The amplified transcript contained a 1137 bp open reading frame and a 142 bp 3′ untranslated region that had a 29 bp polyadenylation tail that began 13 bp downstream from the consensus AATAAA eukaryotic polyadenylation signal (Figure 1A). The transcript was predicted to encode a 378 amino acid protein with a mass of 43.01 kDa and have the domain characteristics of eukaryotic aspartic proteases, including a signal peptide (amino acids 1–19) and an Asp domain (amino acids 65–375) (Figure 1B). The deduced protein also had three potential *N*-glycosylation sites: amino acids 27–29 (NRS), 107–109 (NQS) and 127–129 (NGS) (Figure 1B). To assess the phylogenetic relationships, a neighbor-joining tree was constructed using the *AsAP* protein sequence and aspartic protease proteins from various organisms. The *AsAP* clustered with *Halyomorpha halys* and other hemipteran aspartic proteases that were distantly related to nematode and plant aspartic proteases (Figure 2).

### 3.2. AsAP Is Highly Expressed in the Fat Body and Ovaries of Sexually Mature Females

**Figure 3:**
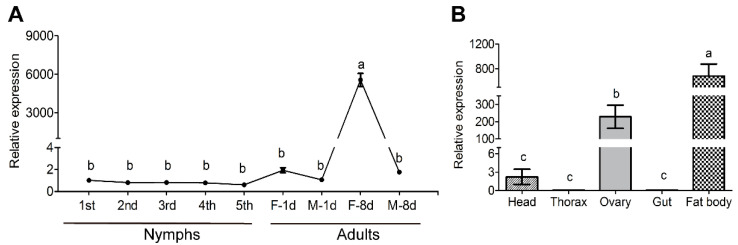
The tissue- and stage-dependent expression profiles of *AsAP* from the RT-qPCR-based analysis of *AsAP* transcript levels across different (**A**) developmental stages or (**B**) tissues. The whole bodies of 1st, 2nd, 3rd, 4th and 5th instar nymphs, sexually immature (1-day-old) male and female adults and sexually mature (8-day-old) male and female adults were collected separately to determine the RNA distribution profiles. Samples from the head, thorax, ovary, gut and fat body were collected separately from 8-day-old females. Elongation factor-1γ (EF1γ) was used as a reference gene for the *AsAP* transcript normalization. The values are expressed as the means ± SEM, based on three independent biological replicates. Different letters show significant differences (*p* < 0.05, one-way ANOVA followed by Tukey’s HSD test).

The elucidation of gene expression patterns can provide insights into their function. Thus, we assessed *AsAP* transcription across different developmental stages and tissues. The *AsAP* expression was highest in 8-day-old females (sexual maturity stage), at which it was nearly three orders of magnitude higher than in the other stages (Figure 3A: F_8,27_ = 121.395, *p* < 0.001). We next analyzed the *AsAP* expression in different tissues (head, thorax, ovary, gut and fat body) from the 8-day-old females. *AsAP* was most abundant in the fat body and high levels were also detected in the ovaries, whereas negligible signals were observed in the thorax and gut (Figure 3B: F_4,10_ = 169.108, *p* < 0.001).

### 3.3. AsAP Is Required for Female Fertility in A. suturalis

**Figure 4:**
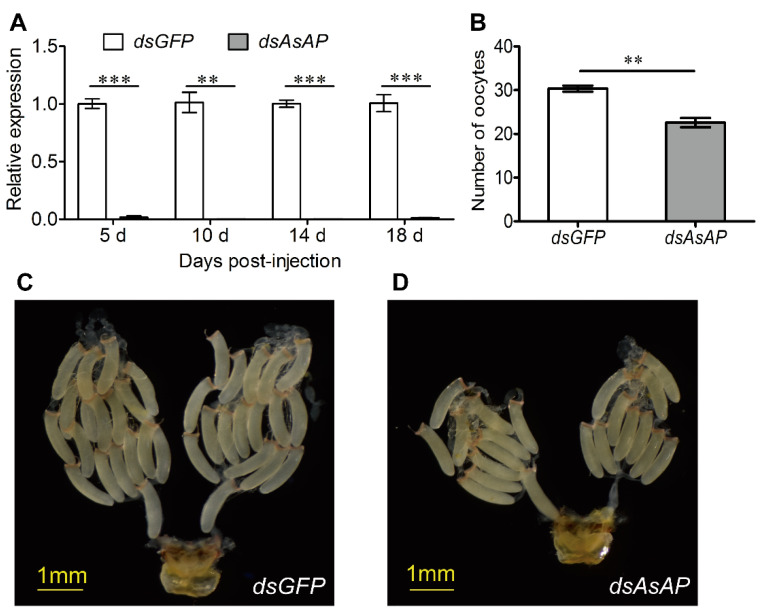
*AsAP* knockdown suppressed ovarian development. Newly emerged females were micro-injected with *dsAsAP* or *dsGFP* (control): (**A**) the *AsAP* transcript levels at 5, 10, 14 and 18 days post-injection; (**B**) the number of oocytes per ovary pair; (**C**) the ovaries from females that were injected with *dsGFP*; (**D**) the ovaries from females that were injected with *dsAsAP*. (**C**,**D**) were imaged at 10 days post-injection using a stereo microscope. All values are expressed as means ± SEM, based on three independent biological replicates. Asterisks indicate statistical significance (** *p* < 0.01 and *** *p* < 0.001, according to Student’s *t*-tests).

**Figure 5:**
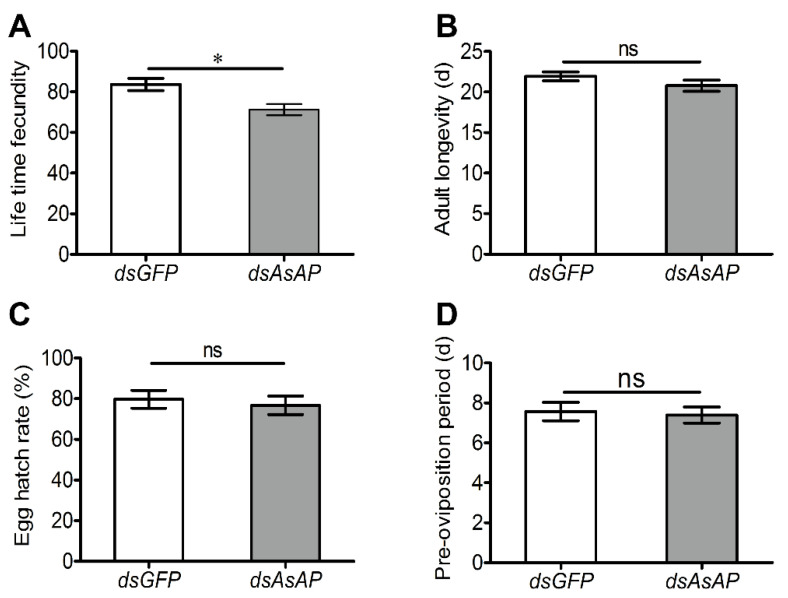
*AsAP* knockdown impacted female fertility. Newly emerged females were micro-injected with *dsAsAP* or *dsGFP* (control) and four reproductive parameters evaluated: (**A**) lifetime fecundity; (**B**) adult longevity; (**C**) egg hatching rate; (**D**) pre-oviposition period. All values are expressed as means ± SEM, based on three independent biological replicates. Asterisks indicate statistical significance (* *p* < 0.05 and ns represents non-significant results, According to Student’s *t*-tests).

Since *AsAP* was upregulated in the ovaries of sexually mature females (i.e., 8-day-old females), we speculated that it could play an important role in *A. suturalis* reproduction. To test this hypothesis, we injected *dsAsAP* into 1-day-old females and monitored their reproductive status over time. Compared to the *dsGFP*-treated control group, the transcript levels of *AsAP* were significantly suppressed at 5, 10, 14 and 18 days post-injection following treatment with *dsAsAP* (Figure 4A: F = 2.690, df = 4, *p* < 0.001; F = 15.076, df = 2, *p* = 0.007; F = 4.159, df = 4, *p* < 0.001; F = 7.513, df = 4, *p* < 0.001). Our assessment of oocyte numbers per ovary pair at 10 days post-injection revealed a 25.6% reduction compared to the control group (Figure 4B: F = 3.289, df = 6, *p* = 0.001). Furthermore, ovarian development was visibly suppressed following the *dsAsAP* injections (Figure 4C,D). A survey of four common reproductive parameters showed that lifetime fecundity was also significantly impacted by *AsAP* knockdown (Figure 5A: F = 0.051, df = 4, *p* = 0.037). In contrast, the other three parameters (pre-oviposition period, adult longevity and egg hatching rate) were comparable to the control group (Figure 5B–D: F = 0.079, df = 6, *p* = 0.786; F = 0.078, df = 6, *p* = 0.241; F = 0.027, df = 4, *p* = 0.659). When taken together, these results indicated that *AsAP* could play an important role in *A. suturalis* ovarian development and fertility.

## 4. Discussion

Given the significant economic impacts that are associated with *A. suturalis* and the emerging pesticide resistance issues, it is clear that new management approaches are needed for pest species. A potential control strategy that has been proposed involves the targeted disruption of *A. suturalis* reproductive capacity. However, neither the physiology nor the molecular underpinnings of *A. suturalis* reproductive development have been sufficiently elucidated. In this study, we isolated a putative *A. suturalis aspartic protease* (*AsAP*)*,* analyzed its expression patterns and assessed its biological function using the RNAi-mediated knockdown method. We found that *AsAP* was highly transcribed in the fat body and ovaries of sexually mature females and that its knockdown significantly reduced both the oocyte number and lifetime fecundity of *A. suturalis* females. No effects were observed on any of the other reproductive parameters that were assessed. Because mature oocyte (egg) formation and, by extension, female fecundity are impacted by the physiology of ovarian development, the observed reduction in female lifetime fecundity could be attributable to reduced rates of oogenesis. However, in this study, we could not fully rule out the potential off-target effects of dsRNA in *A. suturalis*, though we tried to minimize the off-target effects by screening and discarding off-target sites using a bioinformatic analysis. Thus, further validation is still needed.

Vitellogenesis is a major event in insect ovarian development that reportedly involves a variety of proteolytic enzymes, including aspartic proteases [29,30,31,32]. According to current understanding, vitellogenin, inactive acidic proteolytic enzymes and other yolk protein precursors that are primarily produced in the fat body are exported to the maternal hemolymph for uptake by nurse cells into nascent oocytes. The vitellogenin is then stored as vitellin in specialized structures, which are termed yolk bodies. Oocyte maturation proceeds with a concomitant reduction in the yolk protein precursors [29,31,33]. In support of this model, both cathepsin B-like proteinase (vitellogenic cathepsin B) and vitellogenic carboxypeptidase in *Aedes aegypti* are uniquely synthesized in the fat body and incorporated into developing oocytes along with vitellogenin [32,34,35]. It follows that these acid hydrolases are crucial for oocyte development. This was consistent with the phenotype (i.e., reduced numbers of oocytes in developing ovaries) that we observed following *AsAP* knockdown, which suggested that the enzyme was crucial for mature oocyte formation.

Acid hydrolases that are deposited in the ovaries during vitellogenesis have been implicated in the degradation of vitellin [19,36]. For instance, in both *Rhodnius prolixus* and *R. microplus*, aspartic endopeptidases have been shown to play a role in vitellin degradation during embryogenesis [37,38,39]. IrCD3, which is a cathepsin D that is highly expressed in the ovaries of *Ixodes ricinus*, has been shown to have a similar function in yolk protein degradation [8]. In addition, *Dipetalogaster maxima* cathepsin D, which is expressed in ovarian and fat body tissues during the reproductive cycle, has also been proposed as having a function in vitellogenesis [40]. Although these acid hydrolases are usually synthesized outside oocytes, mainly in the fat body, they can also be produced by ovarian follicular cells [8,38,40], which was consistent with the spatial expression profile of *AsAP*. These enzymes are typically stored in an inactive form and are converted into their active form at the onset of embryonic development after eggs have been laid [36,41,42,43]. During the subsequent embryogenesis, the vitellin that is gradually hydrolyzed by proteolytic enzymes is used to support the energetic demands of embryonic development [36]. This raises questions regarding the role of *AsAP*. In our study, the egg hatching rate was not affected by *AsAP* knockdown, which suggested that *AsAP* played a critical role in oocyte formation rather than embryogenesis.

Ovarian development in many insects is usually accompanied by the degeneration of developing follicles or oocytes (i.e., follicular atresia). This process results in some oocytes being resorbed instead of continuing along the typical maturation curve of egg formation [44]. Various physiological and environmental factors can promote follicular atresia and oocyte resorption (oosorption), including poor nutrition, an absence of males, a lack of suitable oviposition sites, overcrowding and low temperatures [44,45]. Oosorption has been proposed as being an adaptive mechanism that balances the conflicting energy requirements of reproduction and survival by recouping resources that may otherwise be lost. These resources can be reinvested into extending the life span and future reproductive potential of the female. In some insects, a characteristic of oosorption is the release of intact vitellin or hydrolytic fragments into the hemolymph [45,46]. Proteolytic enzymes that are involved in yolk protein degradation have also been reported as playing a central role in follicular atresia via vitellin proteolysis. In *D. maxima*, both DmCatD, which is a soluble lysosomal aspartic protease that is expressed in the fat body and ovaries in all reproductive stages, and tyrosine phosphatase are necessary for vitellin degradation [40]. In *Culex pipiens pallens*, cathepsin B and L-like peptidases promote follicular atresia and oosorption by degrading yolk proteins and the follicular structure itself [47]. Similar findings have also been reported in *R. prolixus* [48]. Therefore, we speculated that *AsAP* could affect female fertility by playing a role in oosorption. *AsAP* knockdown could limit the availability of materials in atretic follicles, which could prevent females from recycling the contents for the remaining developing oocytes and could ultimately lead to a decrease in egg production. In the current study, although no increase in the number of ovarian atretic follicles was observed in *dsAsAP*-injected females, these dynamics are often difficult to monitor. The number of normally developing follicles typically exceeds that of follicles that are undergoing degeneration and the process itself, which is very fast, is only evident in advanced stages when most of the oocytes have been resorbed [47,49].

A lack of nutrients that is caused by reduced feeding can also impact insect reproduction [50]. In Hemiptera, aspartic proteases also function in the digestion of ingested compounds, which can impact the energy requirements of biological processes that are vital for survival, development and reproduction [6,7,19,51]. These proteases are typically expressed in midgut cells and/or the salivary gland complex [6,19,52,53,54]. The relative absence of *AsAP* transcripts in the thorax (i.e., the body segment that is associated with the salivary glands) and gut suggests that the effects of *AsAP* on reproduction are not related to diet. Recently, RNAi has been widely used for the study of gene function and furthermore, this technology has become increasingly recognized as a promising and novel pest management strategy. Entomologists use microbe- or plant-mediated RNAi technology to deliver dsRNA into targeted insects feeding on plant tissues and the pests are efficiently killed by the knockdown of critical gene(s) in their growth and development [15,55]. Recently, plant-mediated RNAi technology has been successfully applied in several pest taxa, including *Helicoverpa armigera* [56], *Manduca sexta* [57], aphids [58], *A. suturalis* [28], *Leptinotarsa decemlineata* [59] and more. These findings suggest that plant-mediated RNAi is a feasible and effective strategy for pest control, which has potentially greater levels of environmental safety and pest specificity.

## 5. Conclusions

In summary, we identified an *aspartic protease* gene in *A. suturalis* that was highly expressed in the fat body and ovaries of sexually mature females, which appeared to play an essential role in ovarian development and female fertility. Although the mechanisms through which *AsAP* affected ovarian development remain to be determined, these findings expanded our understanding of *A. suturalis* reproductive development and could provide a potential candidate target gene for development as a novel control strategy.

## Figures and Tables

**Table 1:** The primers that were used for cDNA cloning, RT-qPCR and dsRNA synthesis.

Primer	Primer Sequence (5′–3′)
**5′ RACE**	
B517-1 (GSP1)	tttcttgaggaggatt
B517-2 (GSP2)	atgctgtatgccaccttg
B517-3 (GSP3)	gttccaactcctctatgtaa
**3′ RACE**	
C374-1 (Outer)	cacatttcatatcttgacagacggga
C374-2 (Inner)	tgaaatcgacatcatgggaaagact
**ORF Cloning**	
*AsAP*-F	tgatctaagtgtgatcagtaaagatgattt
*AsAP*-R	gttccataaagtatttaattcgctaaagtcg
**RT-qPCR**	
*AsAP*-F	acccaggcaaccaatccgaa
*AsAP*-R	actgacccattcccgctacc
EF1γ-F	ttggcccttgctgcagaacc
EF1γ-R	tctccgagccagatggagtagtt
**dsRNA Synthesis**	
*dsAsAP*-F	gcgtaatacgactcactataggcccgaacaactgtggtggaa
*dsAsAP*-R	gcgtaatacgactcactataggttggcttggcgatccaaaatc

## Data Availability

The newly sequenced cDNA data are available from GenBank, accession No. OP019357.
